# Human platelet lysate‐derived extracellular vesicles enhance angiogenesis through miR‐126

**DOI:** 10.1111/cpr.13312

**Published:** 2022-08-09

**Authors:** Antonella Bordin, Maila Chirivì, Francesca Pagano, Marika Milan, Marco Iuliano, Eleonora Scaccia, Orazio Fortunato, Giorgio Mangino, Xhulio Dhori, Elisabetta De Marinis, Alessandra D'Amico, Selenia Miglietta, Vittorio Picchio, Roberto Rizzi, Giovanna Romeo, Fabio Pulcinelli, Isotta Chimenti, Giacomo Frati, Elena De Falco

**Affiliations:** ^1^ Department of Medical‐Surgical Sciences and Biotechnologies Sapienza University of Rome Latina Italy; ^2^ Department of Pathophysiology and Transplantation Fondazione IRCCS Cà Granda Ospedale Maggiore Policlinico Milan Italy; ^3^ Institute of Biochemistry and Cell Biology, National Research Council of Italy (IBBC‐CNR) Monterotondo Rome Italy; ^4^ UOC Neurologia, Fondazione Ca'Granda, Ospedale Maggiore Policlinico Milan Italy; ^5^ Institute of Transfusion Medicine and Immunology, Mannheim Institute of Innate Immunoscience, Medical Faculty Mannheim Heidelberg University Mannheim Germany; ^6^ Tumor Genomics Unit, Department of Research IRCCS Fondazione Istituto Nazionale dei Tumori Milan Italy; ^7^ Department of Movement, Human and Health Sciences University of Rome "Foro Italico" Rome Italy; ^8^ Department of Anatomy, Histology, Forensic Medicine and Orthopaedics La Sapienza University of Rome Rome Italy; ^9^ Istituto Nazionale Genetica Molecolare INGM ‘Romeo ed Enrica Invernizzi’ Milan Italy; ^10^ Institute of Biomedical Technologies National Research Council of Italy (ITB‐CNR) Milan Italy; ^11^ Department of Translational and Precision Medicine Sapienza University of Rome Rome Italy; ^12^ Mediterranea Cardiocentro Naples Italy; ^13^ Department of AngioCardioNeurology IRCCS Neuromed Pozzili Italy

## Abstract

**Objectives:**

Extracellular vesicles (EVs) are key biological mediators of several physiological functions within the cell microenvironment. Platelets are the most abundant source of EVs in the blood. Similarly, platelet lysate (PL), the best platelet derivative and angiogenic performer for regenerative purposes, is enriched of EVs, but their role is still too poorly discovered to be suitably exploited. Here, we explored the contribution of the EVs in PL, by investigating the angiogenic features extrapolated from that possessed by PL.

**Methods:**

We tested angiogenic ability and molecular cargo in 3D bioprinted models and by RNA sequencing analysis of PL‐derived EVs.

**Results:**

A subset of small vesicles is highly represented in PL. The EVs do not retain aggregation ability, preserving a low redox state in human umbilical vein endothelial cells (HUVECs) and increasing the angiogenic tubularly‐like structures in 3D endothelial bioprinted constructs. EVs resembled the miRNome profile of PL, mainly enriched with small RNAs and a high amount of miR‐126, the most abundant angiogenic miRNA in platelets. The transfer of miR‐126 by EVs in HUVEC after the in vitro inhibition of the endogenous form, restored angiogenesis, without involving VEGF as a downstream target in this system.

**Conclusion:**

PL is a biological source of available EVs with angiogenic effects involving a miRNAs‐based cargo. These properties can be exploited for targeted molecular/biological manipulation of PL, by potentially developing a product exclusively manufactured of EVs.

## Graphical abstract

A high amount of small‐size extracellular vesicles (EVs) can be isolated from platelet lysate (PL)‐based preparations. When endothelial cells (HUVEC) are cultured in presence of EVs of platelet origin, they are able to significantly enhance the formation of angiogenic tubularly‐like structures in 3D endothelial bioprinted constructs (2A,B). PL‐derived EVs reflect a similar angiogenic microRNA profile (3A). Accordingly, EVs are mainly enriched with miR‐126, also known as angio‐miRNA, the most expressed miRNA by platelets in the blood. Hence, the silencing of the endogenous levels of miR‐126 in HUVEC and the retransferring of the same through PL‐derived EVs, restore angiogenesis in endothelial cells (3B). Images were created with the Biorender software.

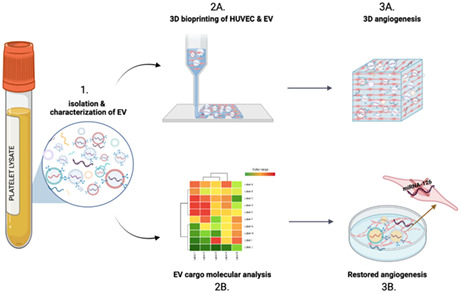

## 1 INTRODUCTION

Beyond haemostasis and thrombosis, platelets have been also described as the main regulators of angiogenesis, a key process for tissue regeneration and repair outcome of vascular insults or wound healing and based on the activation of endothelial proliferation, sprouting and organization into functional tubules.^1^, ^2^, ^3^ The emerging role of platelets to act as inflammatory/immune effectors and to enhance angiogenesis, stems from their intrinsic physiological role to interact with the endothelium during vascular damage, preserving the integrity and vessel homeostasis.^4^

Platelets exhibit a unique secretory profile of multiple combined factors with a dual pro‐ and anti‐angiogenic role. Among them, we could list growth factors, cytokines, microRNAs, small soluble molecules and proteins, including those related to cytoskeleton, adhesion, inflammation, and extracellular matrix interaction.^5^, ^6^, ^7^, ^8^ This balanced combination of mediators, is mainly contained in plasma membranes delimited nanoparticles, named extracellular vesicles (EVs). These later, now conceived as signalosomes and biological vectors of heterogeneous size and composition, are released upon platelets activation, interacting with the microenvironment.^9^, ^10^

Platelets represent the most abundant source of EVs of different dimensions and quantities in the systemic circulation^11^, ^12^, ^13^, ^14^ depending on multiple variables including age, physiological states and lifestyle habits.^15^, ^16^ EVs mirror the haemostatic properties of platelets,^17^ by exerting both anti‐ and pro‐coagulant effects according to the subpopulation of EVs involved.^11^, ^18^, ^19^ Hence, their circulating levels can act as predictive biomarkers of haemostatic and inflammatory disorders.^20^ Patients with metabolic syndrome, myocardial infarction, atherosclerosis, ischemia or inflammatory diseases exhibit higher levels of circulating EVs, because of activated platelets,^21^, ^22^, ^23^, ^24^, ^25^, ^26^, ^27^, ^28^ suggesting their relevance to mediate pathogenetic effects beyond their physiological role. On the other hand, platelet‐derived EVs have been also demonstrated to regulate angiogenesis when released at the site of endothelial sprouts^29^ and secretion of VEGF,^30^ or to transfer proliferative and survival biological information to the endothelium.^30^, ^31^, ^32^, ^33^ Platelet‐derived EVs can modulate the vascular tone as shown in rabbit models,^30^ or even attenuate blood pressure in preeclampsia women, by stimulating the inducible nitric oxide synthase in endothelial cells,^34^ consistently with a beneficial functional role on the vasculature.

Similarly to their parental cells, EVs would also act indirectly in a paracrine fashion on the intercellular network as cargos of cytokines and decoy proteins are locally released in the microenvironment. For instance, endothelial progenitor cell‐mediated angiogenesis^1^, ^3^ and tissue engraftment is enhanced by EVs of platelets origin through the activation of specific targets (MMP‐2, MMP‐9, PI3K, ERK) or after the transfer of specific soluble platelet receptors and activation of integrins on the endothelial surface.^30^, ^35^, ^36^, ^37^ The preconditioning of bone marrow mesenchymal stem cells (MSCs) with platelet‐derived EVs has demonstrated their effective capacity of boosting the biological potency and vascular effects of the stromal fraction.^38^ Strong evidence of their ability to support angiogenesis has been also observed in myocardial infarction and cerebral ischemia after in vivo direct injection, when platelets, activated by thrombin, release EVs.^39^, ^40^ Moreover, the key contribution of platelet‐derived EVs in supporting the angiogenic profile of cancer invasion and metastasis parallel to clinical thrombotic complications has been strongly highlighted.^41^, ^42^, ^43^

Based on these studies, evidence that platelets and platelet‐derived biological products can trigger angiogenic programs in endothelial cells has encouraged a better understanding of their potential therapeutic use for those regenerative‐based applications where the restoration or the enhancement of angiogenesis represents the clinical goal. Accordingly, parallel to the investigations regarding the key involvement of platelets in regulating angiogenesis, it has been demonstrated that platelet‐derived clinical preparations (i.e., platelet‐rich plasma and gels) are similarly able to boost and reflect the angiogenic properties of platelets. Particular attention has been dedicated to platelet lysate (PL), considered the gold preparation concentrate derived from platelets, and whose clinical efficacy is currently considered superior to other platelet‐derived formulations.^44^ The employment of PL, alone or even in combination with different sources of stem cells, has shown to enhance blood perfusion in peripheral artery diseases,^45^ to heal difficult wounds, to sustain stromal proliferation, epithelization, angiogenesis, and to prime cardiovascular differentiation.^1^, ^2^, ^3^, ^46^, ^47^, ^48^, ^49^ The angiogenic capacity of PL is ascribable to the plethora of highly concentrated factors in this hemoderivative. When PL is manufactured, platelets are repeatedly lysed, therefore enriching the preparations with vesicles and granules, representing a primary source of angiogenic EVs. So far, the vast majority of studies have only explored the effects of vesicles of different origins (i.e., from MSCs, fibroblasts, lymphocytes) after treatment with PL, or EVs released by intact and activated platelets.^43^ Only a couple of studies have described the presence of exosomes in platelet‐derived clinical formulations but as effectors of the osteogenic differentiation on MSCs or with neuroregenerative capacities.^38^, ^50^ Thus, the wide range of mechanisms by which PL‐derived EVs might regulate angiogenesis still needs to be fully addressed.

This study investigates from a biological and molecular standpoint the role of EVs in relation to PL formulations regarding the ability to mediate angiogenesis by endothelial cells.

## 2 METHODS

### 2.1 Isolation of EVs from PL‐based preparations

EVs were isolated from PL‐based preparations^2^, ^46^, ^47^, ^48^, ^49^, ^51^ by sequentially ultracentrifugation (500 rcf for 10 min; 2000 rcf for 10 min; 100,000 rcf for 1 h). EV pellet was then resuspended to reconstitute the initial PL volume and subsequently used at 5%, 10% or 20% in the medium for the experiments. For FACS analysis and uptake assay in human umbilical vein endothelial cell (HUVEC), 100 μl of EVs were stained for 10 min at 37°C with 5 μM 5(6)‐CFDA‐SE [5‐(and‐6)‐carboxyfluorescein diacetate, succinimidyl ester] (CFSE; Invitrogen/Thermo Fischer Scientific) according to the manufacturer's instructions. Excess dye was removed using Exosome Spin Columns (Thermo Fischer Scientific) following the manufacturer's recommendations.

### 2.2 Nanotracking analysis of EVs in PL

Nanoparticles tracking analysis in terms of size distribution and concentration was performed on PL using a NanoSight NS300 instrument (Malvern Panalytical). Five 30‐s videos were recorded for each sample with a camera level set at 15 of 16 and a detection threshold set between 5 and 7. The EVs concentration and size distribution were subsequently analysed with NTA 3.2 software.

### 2.3 Western blot

EV were resuspended in a radioimmunoprecipitation buffer and phosphatase inhibitor cocktail. Proteins (10 μl solubilized in 2X Laemmli/20% of 2‐mercaptoethanol) were separated by sodium dodecyl sulphate–polyacrylamide gel electrophoresis on 10% polyacrylamide gel (Bio‐Rad). Subsequently, the membrane was blocked with 5% (wt/vol) milk and the membranes were incubated at 4°C overnight with rabbit Anti‐Annexin A1/ANXA1 antibody monoclonal antibody [EPR19342]‐BSA and Azide free (1:25,000; Abcam; Cat. N. ab222398), ALIX (1:1000; Biorbyt; Cat. N. orb235075), CD9 (1:500; Abcam; ab186429), calnexin (1:1000; Santa Cruz; Cat. N. sc‐46669), TSG101 (4A10) (1:500; Invitrogen; Cat. N. #MA1‐23296) After incubation, the membranes were incubated with secondary anti‐rabbit antibody (Cell Signalling; 1:10,000) and the immune complexes thus formed were detected by enhanced substrate chemiluminescence. Densitometric detection of the bands was performed by Chemidoc (Bio‐Rad).

### 2.4 Cell culture, transfection and treatment with antagomiR‐126

HUVECs were cultured between passages 3 and 6 in an EGM‐2 complete medium (Lonza).^3^, ^52^, ^53^, ^54^, ^55^ Fluorescein‐conjugated antagomirs were used for quantification of antagomir or control incorporation and detected by flow cytometry. For antagomiR‐126 transfection, cells were plated at a density of 3.5 × 10^4^/24 wells with EGM‐2 without gentamicin. A mix composed of 25 pmol LNA_126 (miRCURY LNA miRNA Inhibitor (5) ‐ 3′ Fam; Cat. N. 339121; Qiagen) or 25 pmol control (miRCURY LNA miRNA Inhibitor Control (5)‐No Modification Fam; Cat N. 339126; Qiagen) in Opti‐MEM Reduced‐Serum Media and lipofectamine (1 μl/100 μl Optimem; RNAiMAX; Invitrogen; Cat. N. 56531) was added to the HUVEC and incubated overnight. The next day, the medium was removed, and new EGM‐2 was added to the cells for up to 24 and 48 h of total transfection. To verify transfection, HUVEC were analysed by flow cytometry detecting FAM fluorescence. See Supporting Information Methods S1, for cytometric analysis, transmission electron microscopy, Matrigel assay and immunofluorescence on HUVEC in 3D‐bioprinting constructs, platelets aggregation, determination of soluble human prothrombin fragment 1 + 2 and quantification of peroxide hydrogen and molecular biology.

### 2.5 Statistics

Statistical analysis was performed by GraphPad PRISM 5 software. Student's *t*‐test and one‐way analysis of variance (Bonferroni correction) were used to compare the difference between the control and groups. A *p* < 0.05 was considered significant. Data were presented as mean ± standard error unless specified. Additional information on statistics and confidence intervals have been reported in the corresponding sections above and in figure legends.

## 3 RESULTS

**FIGURE 1:**
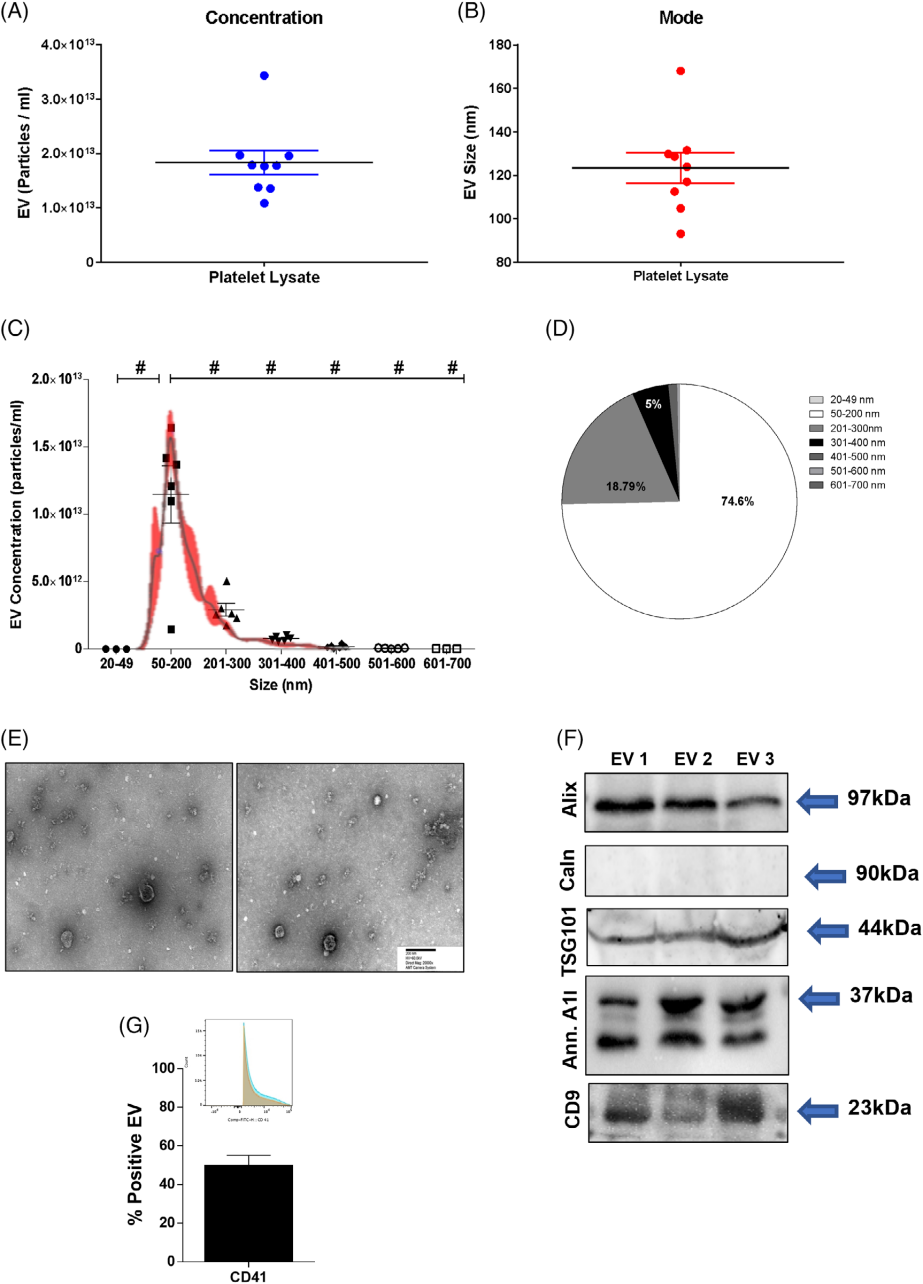
Characterization of extracellular vesicles (EVs) in platelet lysate (PL). (A) Concentration measurement and (B) size mode from Nanoparticle Tracking Analysis (NTA) of EVs derived by nine batches of PL. (C) NTA graph and (D) pie chart display the EVs size distributions. ^#^ *p* < 0.001. (E) Representative transmission electron microscopy images of EVs within PL‐based preparations. Scale bar (200 nm) and magnification (×20,000) are displayed. (F) Western blot analysis of ALIX, calnexin, TSG101, Annexin A1 and CD9 of three EV batches isolated by ultracentrifugation from PL. (G) Representative histogram of the flow cytometry displaying the relative percentage of EVs in the graph below for CD41, platelet marker. *N* = 3 lots of EVs were tested

We investigated in detail the EV content and characteristics of human PL preparations. To assess the concentration and absolute size distribution,^56^ we measured the EVs by Nanoparticle Tracking Analysis (NTA) in eight different batches of PL. Results showed that PL‐based preparations contain a very high concentration of EVs with a mean of 1.84 × 10^13^ particles/ml, with the EV size mode of 123.37 ± 7.02 nm (Figure 1A,B). Among all distinctive subclasses of EVs, the most representative group in terms of concentration was the 50–200 nm subset, corresponding to small microvesicles including exosomes^57^ (Figure 1C,D, *p* < 0.001 vs. all subsets). Occasionally, EVs of <50 nm size were found, but not in all batches.

Following international guidelines,^58^, ^59^ EVs were further characterized by evaluating their morphology and phenotype by transmission electronic microscopy (TEM), and Western blot for the recommended universal markers. TEM analysis confirmed that PL preparations contained EVs with heterogeneous but small dimensions, roundish morphology and electron‐dense features, suggesting a significant cargo function (Figure 1E). According to the Minimal Information for Studies of Extracellular Vesicles (‘MISEV’) guidelines,^58^ TEM was qualitatively implemented by both Western blot showing the positive expression of the proteins ALIX, CD9, Annexin A1, TSG101 (cytosolic, membrane and marker of biogenesis of EV) in PL‐derived EVs^60^, ^61^ and negative expression for calnexin, therefore suggesting the absence of non‐EV structures in the preparation of EVs^62^ (Figure 1F). The characterization of EVs was further verified by cytofluorimetry. The FACS analysis confirmed the expression of CD41, the main marker of platelet origin of EVs (49.92 ± 5.22%, also known as glycoprotein IIb possessing a critical role in modulating platelet aggregation^63^), but also the negative expression for calnexin (Figure 1G).

**FIGURE 2:**
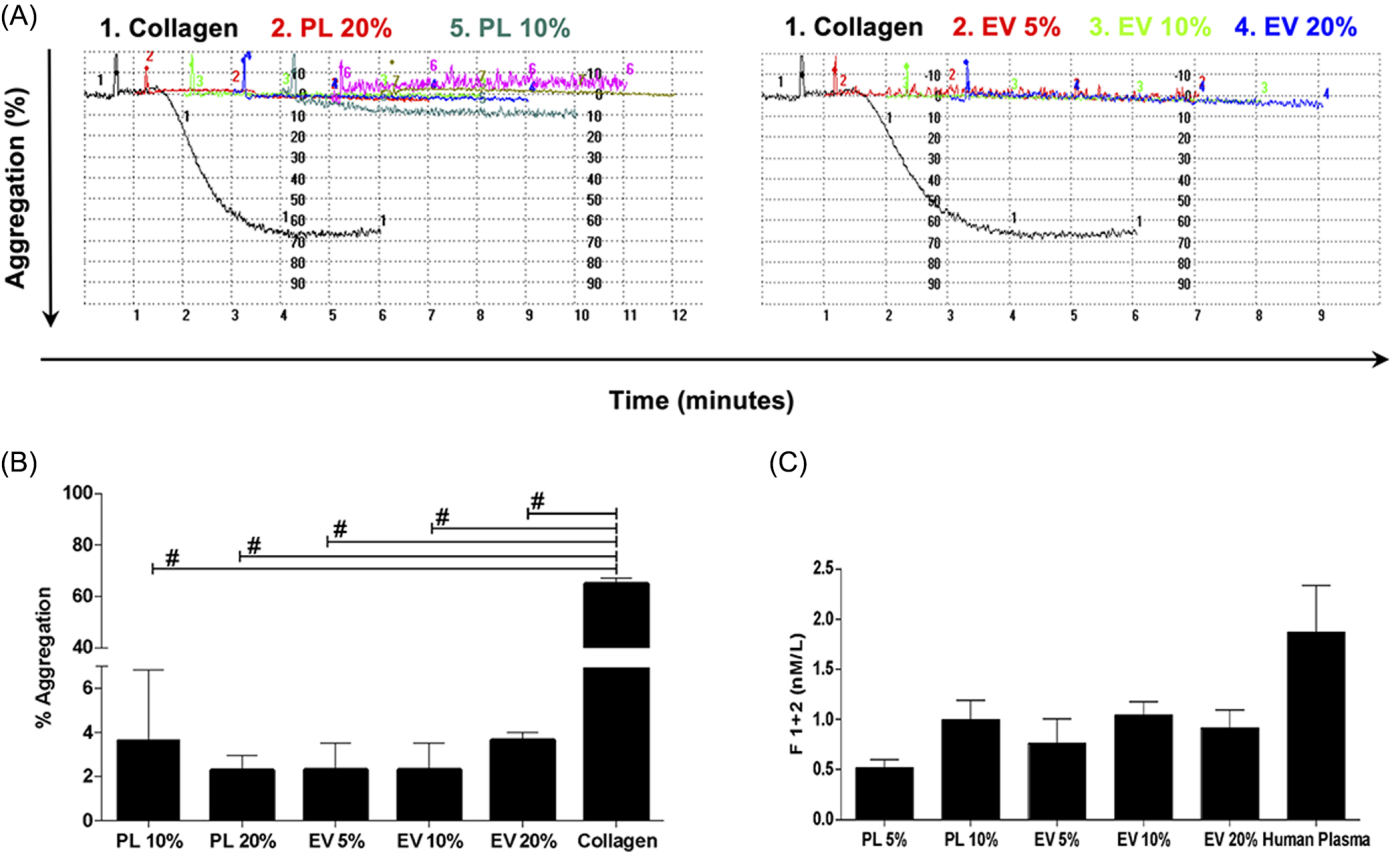
Hemostatic properties of EVs derived from PL. (A) Representative plots of the aggregometer displaying both PL and EVs at different percentage and (B) relative analysis showing no aggregation compared to collagen used as positive reference. ^#^ *p* < 0.001 (C) Immunoassay of the F1 + 2 assaying the coagulative ability of both PL and EVs compared with human plasma with no significant difference. EV, extracellular vesicle; PL, platelet lysate

To discriminate the biological effects of EVs from the whole PL, we isolated the EVs according to methodological standardized guidelines by high‐speed ultracentrifugation.^64^, ^65^ Afterwards, we investigated whether EVs may convey haemostatic properties, such as aggregation and pro‐coagulant abilities, which are two key physiological properties exerted by platelets but also reported for EVs.^66^ We stimulated platelets of healthy subjects with increasing percentages of EVs (5%, 10% and 20%). PL (10% and 20%) and collagen were used as biological and positive references, respectively. In addition, the quantification of the soluble fragment 1 + 2 of prothrombin (F1 + 2) was employed to test the coagulation property of EVs. Results showed that neither the increasing concentration of EVs nor PL were able to induce aggregation compared to collagen (Figure 2A,B). A similar amount of F1 + 2 among samples was detected (comparable to physiological soluble levels in the human plasma), with no statistically significant differences (Figure 2C).

**FIGURE 3:**
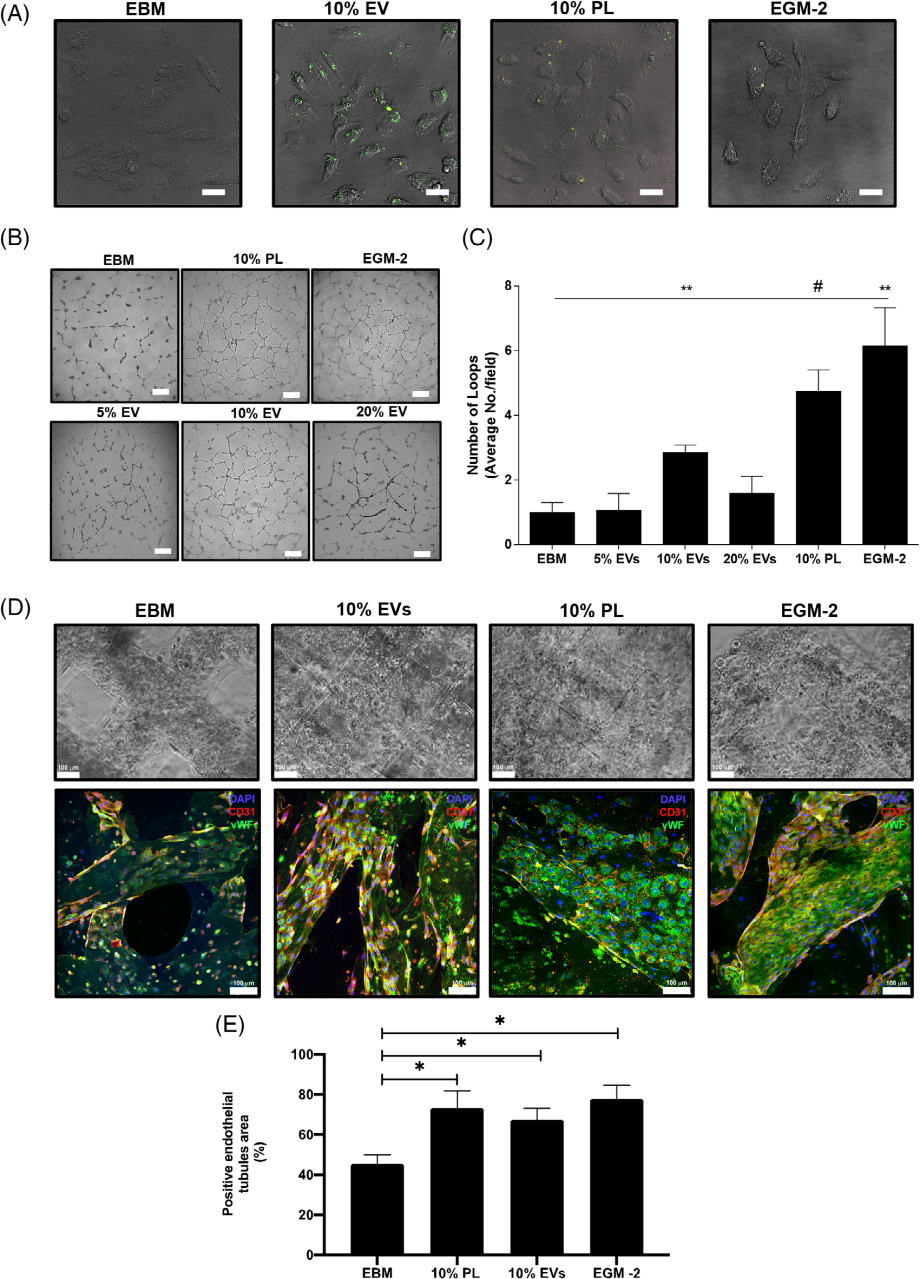
Angiogenic effects of PL‐derived EVs in endothelial cells. (A) Merged images of optical and fluorescent microscopy showing HUVEC uptaking after 24 h the CSFE‐labelled isolated EVs in different experimental conditions (EBM and EGM‐2 negative and positive controls, respectively). White scale bar = 50 μm. (B) Representative images of capillary‐like structures from Matrigel assay and (C) quantitative analysis of number of formed loops with different percentage of EVs (5%, 10% and 20%). Magnification ×4. White scale bar = 200 μm. *p < 0.05, **p < 0.01, ^#^ *p* < 0.001. One‐way ANOVA test was applied. A range of *N* = 3–12 experiments was performed. (D) Confocal microscopy images of 3D in vitro bioprinted HUVEC (bright and fluorescent images), displaying the formation of 3D‐tubules angiogenic structures. DAPI, CD31 and vWF stain blue, red and green, respectively. White scale bar = 100 μm. (E) Analysis of the immunofluorescence indicating the percentage of the positive 3D endothelial tubules area in the different conditions. *p < 0.05. ANOVA, analysis of variance; EV, extracellular vesicle; HUVEC, human umbilical vein endothelial cell; PL, platelet lysate

As one of the most significant bioactive properties of PL is the ability to induce angiogenesis,^2^, ^46^, ^47^, ^48^, ^49^ we investigated the contribution of EVs to the angiogenesis stimuli mediated by PL. We isolated and labelled the EVs with the green fluorescent dye CFSE. Afterwards, HUVECs were stimulated for 24 h with the EV preparation (10% vol/vol, corresponding to the same PL volume in percentage routinely employed in cell culture^47^, ^48^, ^49^, ^51^). HUVECs were able to uptake EVs, as demonstrated by the presence of green fluorescent dots visible in the cytoplasm (Figure 3A). When HUVECs were subjected to the in vitro angiogenesis Matrigel assay at increasing concentrations of EVs (5%, 10%, 20%), we found that 10% EVs was the optimal percentage to significantly enhance the number of closed loops (Figure 3B,C) compared to the negative control (EBM, non‐supplemented endothelial basal media, *p* = 0.0038). Platelets lysate and EGM‐2 were used as positive angiogenic inducers. To corroborate this observation, we employed a 3D bioprinting‐based approach by encapsulating HUVECs in a gelatin/methacrylamide (GelMA) bioink, to evaluate their ability to induce the generation of vessel‐like structures in a more physiologically suitable 3D microenvironment in presence of 10% EVs (the best performer in the Matrigel assay). The confocal microscopy analysis showed that endothelial cells were able to colonize the bioprinted construct after treatment with both PL and PL‐derived EVs. Coherent with the observed spatial distribution, the proportion of the endothelial area (defined as CD31^+^/vWF^+^), corresponding to the organization of HUVECs in 3D tubular structures, was significantly higher with PL‐derived EV and PL treatments, compared to EBM control (Figure 3D,E; *p* < 0.05 vs. 10% EVs and 10% PL).

**FIGURE 4:**
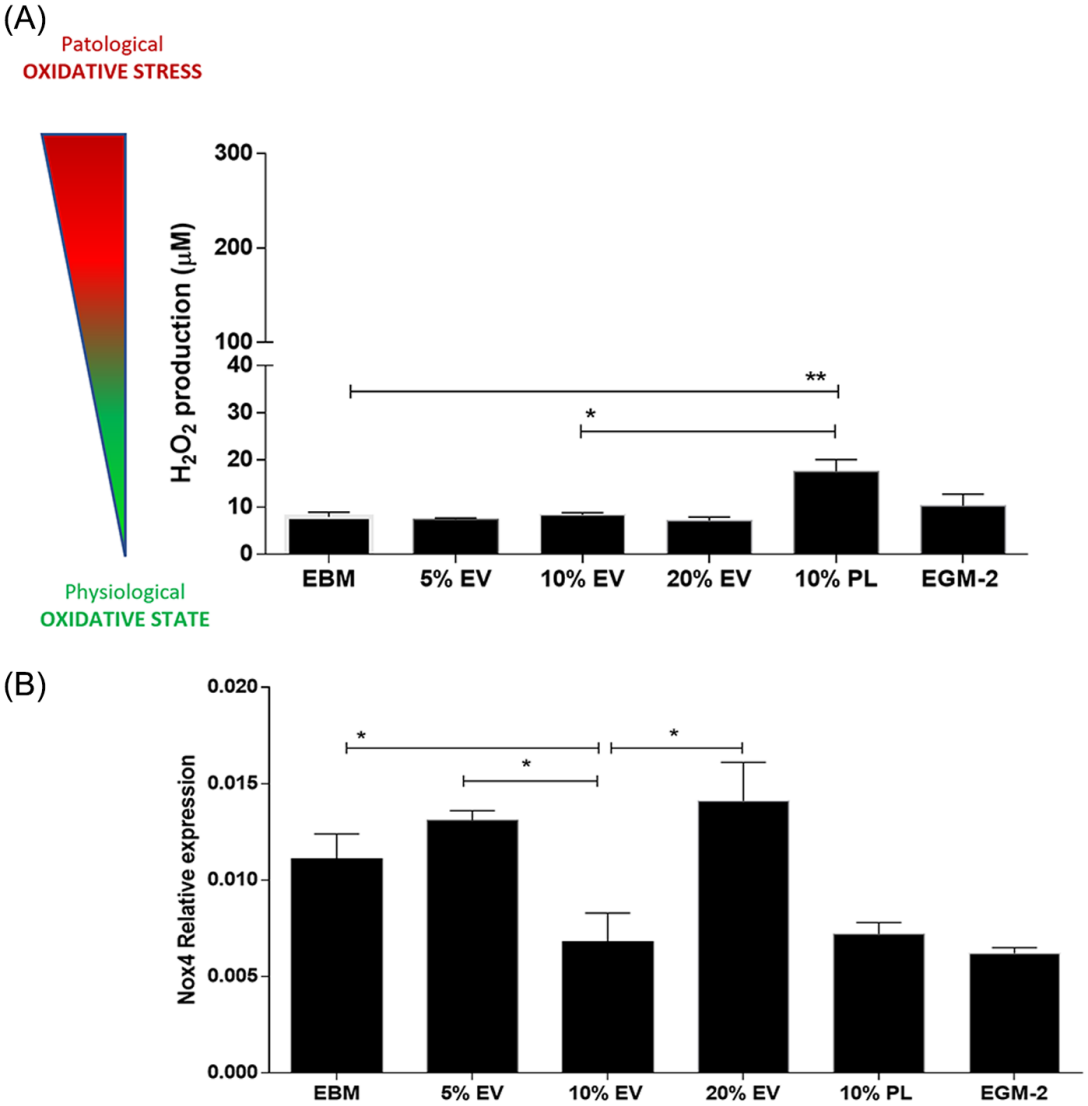
Analysis of the oxidative states of endothelial cells after treatment with different percentage of PL‐derived EVs (5%, 10%, 20%). (A) Hydrogen peroxide (H_2_O_2_) measurement of HUVEC‐derived condition media in all experimental conditions. The cartoon on the left of the graph shows the wide concentration range of H_2_O_2_ from physiological states (≤100 μM) to oxidative stress (≥300 μM). *p < 0.05, **p < 0.01. One‐way ANOVA test was applied. A range of *N* = 3–16 experiments was performed. (B) Relative expression of the NADPH oxidase isoform 4 (NOX4) assayed by real‐time PCR and downregulated in HUVEC after treatment with 10% EVs respect to EBM and all other percentage of EVs (5% and 20%). The effect is also and comparable to that exerted by 10% PL and EGM‐2. *p < 0.05. One‐way ANOVA test was applied. A range of *N* = 3–11 experiments was performed. ANOVA, analysis of variance; EV, extracellular vesicle; HUVEC, human umbilical vein endothelial cell; PL, platelet lysate

Several studies have demonstrated the modulation of the redox status in cells exposed to intact platelet‐derived EVs.^67^ Thus, we investigated the levels of hydrogen peroxide in the conditioned media of HUVECs collected after 24 h of treatment with EVs. Results showed a lower release of hydrogen peroxide after treatment with 10% EVs compared to PL (*p* = 0.03; Figure 4A). We observed that the treatment with all percentages of EVs were able to maintain very low amounts of H_2_O_2_ in the media as both controls (EBM and EGM‐2). However, the 10% EVs reveals as the optimal anti‐oxidant stimulation respect to 10% PL (*p* < 0.05). This result was also coherent with the lowest expression level of the NADPH isoform Nox4 (the main and specific isoform responsible for the direct production of hydrogen peroxide by endothelial cells^52^, ^53^, ^68^, ^69^, ^70^) after stimulation with 10% EVs among the three concentrations of EVs (*p* < 0.05; Figure 4B). The Nox4 mRNA levels in presence of 10% EVs were similarly downregulated as PL and EGM‐2 with respect to EBM (*p* < 0.05).

**FIGURE 5:**
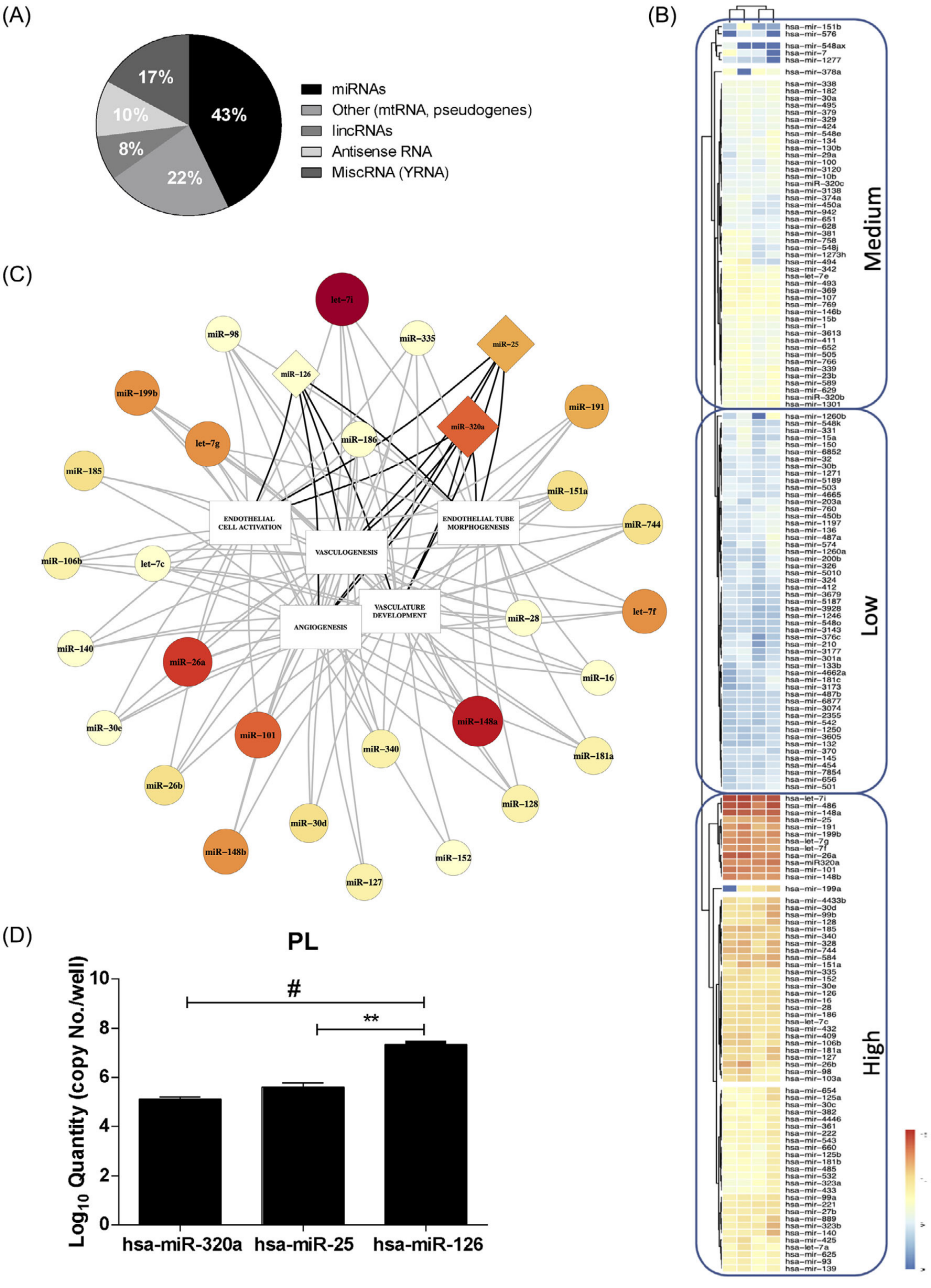
MiRNome characterization of platelet lysate by small RNA sequencing. (A) Small RNA percentage distribution in PL (a total of four replicates). (B) The heatmap has been obtained by including highly and consistently expressed miRNAs among the four analysed replicates with a cut‐off of >10 copies. The red‐yellow and the blue range colour indicate miRNAs with high and low average copy count, respectively. (C) Integration of the biological function with the expression of miRNAs in PL using a linkage group intertwined with five GO terms. Both the dimension of the box (circle/triangle) and the colour range reflect the expression levels as in the heatmap. (D) Quantitative PCR of hsa‐miR‐hsa‐320a, hsa‐miR‐25 and hsa‐miR‐126 have been employed to quantitatively validate the miRNome of PL. These three miRNAs are represented in the triangle box in C. PL, platelet lysate

Some key functions of platelets, such as aggregation, activation and angiogenesis, are known to be mediated by miRNAs released by platelets in response to a wide range of stimuli, both physiological and pathological.^71^, ^72^ This ability can also be mediated by EVs, since they are known to transfer information to target cells through miRNAs,^73^ and therefore to determine diverse biological effects in relation to the cargo within the vesicles. With these premises, we hypothesized the presence of miRNAs in PL‐based formulations and assessed this by analysing the miRNA profile of two different batches of PL for a total of four replicates. Results showed that the majority of the small RNA content in PL is represented by miRNAs (43%), followed by Y RNAs (17%), anti‐sense RNAs (10%), and lincRNAs (8%) (Figure 5A). A miscellaneous group is also represented (22%). After applying a cut‐off of >10 copies in all analysed batches of PL (average count among the four PL samples; Table 1), the identified miRNAs clustered into three macrogroups based on their expression levels (low, medium and high) when analysed by heatmap with hierarchical clustering (Figure 5B).

**TABLE 1:** List of all miRNAs contained in PL and obtained by small RNA sequencing Illumina

ENSEMBL gene	miRBase ID	Avg count (four replicates)
ENSG00000199179	hsa‐let‐7i	35,420.48575
ENSG00000283450.1	hsa‐miR‐486	25,404.28176
ENSG00000199085	hsa‐miR‐148a	21,102.40537
ENSG00000207789.1	hsa‐miR‐26a	16,213.89862
ENSG00000208037	hsa‐miR320a	9701.952607
ENSG00000199065.3	hsa‐miR‐101	9436.507077
ENSG00000199122	hsa‐miR‐148b	7450.524382
ENSG00000199150	hsa‐let‐7g	7267.479277
ENSG00000207581	hsa‐miR‐199b	6969.810643
ENSG00000208012	hsa‐let‐7f	6357.87107
ENSG00000207605	hsa‐miR‐191	5812.577567
ENSG00000207547	hsa‐miR‐25	5236.777868
ENSG00000207948	hsa‐miR‐328	3040.629801
ENSG00000207714	hsa‐miR‐584	2989.181797
ENSG00000266297	hsa‐miR‐744	2822.249642
ENSG00000254324	hsa‐miR‐151a	2812.521101
ENSG00000199121	hsa‐miR‐26b	2632.304808
ENSG00000208023	hsa‐miR‐185	2630.517199
ENSG00000199153	hsa‐miR‐30d	2122.871247
ENSG00000207550	hsa‐miR‐99b	2005.789909
ENSG00000198995	hsa‐miR‐340	1944.070041
ENSG00000199107	hsa‐miR‐409	1709.570737
ENSG00000207595.1	hsa‐miR‐181a	1664.90131
ENSG00000207654.4	hsa‐miR‐128	1615.027686
ENSG00000264297	hsa‐miR‐4433b	1526.263215
ENSG00000208036	hsa‐miR‐106b	1475.271861
ENSG00000207608	hsa‐miR‐127	1461.537107
ENSG00000208004	hsa‐miR‐323b	1379.391397
ENSG00000207651	hsa‐miR‐28	1345.078854
ENSG00000208017	hsa‐miR‐140	1321.751562
ENSG00000271886	hsa‐miR‐98	1222.679492
ENSG00000199030	hsa‐let‐7c	1214.65648
ENSG00000272458	hsa‐miR‐432	1207.070114
ENSG00000199161	hsa‐miR‐126	1171.006656
ENSG00000207721	hsa‐miR‐186	1166.72288
ENSG00000207947	hsa‐miR‐152	1111.222163
ENSG00000198987	hsa‐miR‐16	1072.827202
ENSG00000216099	hsa‐miR‐889	1046.656644
ENSG00000198974	hsa‐miR‐30e	1001.082556
ENSG00000199043	hsa‐miR‐335	982.9147665
ENSG00000199024.1	hsa‐miR‐103a	974.3511292
ENSG00000207752	hsa‐miR‐199a	919.4531345
ENSG00000207870	hsa‐miR‐221	895.0699612
ENSG00000207864	hsa‐miR‐27b	821.4878295
ENSG00000199032	hsa‐miR‐425	772.8305776
ENSG00000207638	hsa‐miR‐99a	740.1333674
ENSG00000207934	hsa‐miR‐654	621.1900075
ENSG00000208008	hsa‐miR‐125a	597.3963088
ENSG00000207725	hsa‐miR‐222	538.5877917
ENSG00000198975	hsa‐let‐7a	515.1025809
ENSG00000207781	hsa‐miR‐625	505.7290621
ENSG00000265253	hsa‐miR‐4446	451.4169995
ENSG00000212040	hsa‐miR‐543	409.0533201
ENSG00000207757	hsa‐miR‐93	403.8187224
ENSG00000272036	hsa‐miR‐139	399.12804
ENSG00000207962	hsa‐miR‐30c	390.0986257
ENSG00000199051	hsa‐miR‐361	389.718269
ENSG00000207970	hsa‐miR‐660	340.2597669
ENSG00000207758	hsa‐miR‐532	318.869568
ENSG00000208008.1	hsa‐miR‐125b	315.48886
ENSG00000283170	hsa‐miR‐382	314.8899174
ENSG00000207737.1	hsa‐miR‐181b	314.5318373
ENSG00000208027	hsa‐miR‐485	289.3005292
ENSG00000211580	hsa‐miR‐769	283.8908085
ENSG00000199025	hsa‐miR‐369	273.869608
ENSG00000199069	hsa‐miR‐323a	248.201048
ENSG00000198972	hsa‐let‐7e	228.5801569
ENSG00000207569	hsa‐miR‐433	218.3957338
ENSG00000207989	hsa‐miR‐493	216.51903
ENSG00000202569	hsa‐miR‐146b	214.64207
ENSG00000198997	hsa‐miR‐107	203.1544061
ENSG00000194717	hsa‐miR‐494	200.0772964
ENSG00000199023	hsa‐miR‐339	193.5326846
ENSG00000199082	hsa‐miR‐342	190.9249656
ENSG00000221406.1	hsa‐miR‐320b	180.4782364
ENSG00000221445	hsa‐miR‐1301	166.0596828
ENSG00000207633	hsa‐miR‐505	149.7697394
ENSG00000207563	hsa‐miR‐23b	149.2282785
ENSG00000207973	hsa‐miR‐589	143.1520471
ENSG00000208013	hsa‐miR‐652	133.0130015
ENSG00000211578	hsa‐miR‐766	129.7966503
ENSG00000207965	hsa‐miR‐629	125.3803452
ENSG00000207779	hsa‐miR‐15b	125.0636251
ENSG00000199047	hsa‐miR‐378a	121.8129584
ENSG00000199020	hsa‐miR‐381	116.4263774
ENSG00000199017	hsa‐miR‐1	109.082889
ENSG00000199109	hsa‐miR‐411	103.8221213
ENSG00000264864	hsa‐miR‐3613	97.42976184
ENSG00000283604	hsa‐miR‐338	94.82506088
ENSG00000221214	hsa‐miR‐548e	92.96228251
ENSG00000211582	hsa‐miR‐758	91.70920684
ENSG00000207990	hsa‐miR‐182	90.79786569
ENSG00000207993	hsa‐miR‐134	88.31512911
ENSG00000283871	hsa‐miR‐130b	83.90167079
ENSG00000207827	hsa‐miR‐30a	81.14539472
ENSG00000221760	hsa‐miR‐548j	77.90232379
ENSG00000207743	hsa‐miR‐495	76.96521629
ENSG00000266192	hsa‐miR‐1260b	74.37795737
ENSG00000207703	hsa‐miR‐7	74.26392405
ENSG00000199088	hsa‐miR‐379	72.24067434
ENSG00000207762.1	hsa‐miR‐329	71.91954104
ENSG00000284032	hsa‐miR‐29a	68.38616085
ENSG00000274466	hsa‐miR‐1273 h	66.08291135
ENSG00000284231	hsa‐miR‐424	65.47781571
ENSG00000199168	hsa‐miR‐374a	60.16913084
ENSG00000207994	hsa‐miR‐100	58.7905659
ENSG00000207744	hsa‐miR‐10b	55.09601822
ENSG00000221493.1	hsa‐miR‐320c	54.59647569
ENSG00000264931	hsa‐miR‐3138	53.8840101
ENSG00000265154	hsa‐miR‐151b	53.70004073
ENSG00000283152	hsa‐miR‐3120	52.16606838
ENSG00000207742	hsa‐miR‐487a	50.73182344
ENSG00000207628	hsa‐miR‐651	50.33440178
ENSG00000199172	hsa‐miR‐331	46.95913882
ENSG00000207782	hsa‐miR‐150	46.70553224
ENSG00000207755	hsa‐miR‐450a	43.8147299
ENSG00000283891	hsa‐miR‐628	41.5096656
ENSG00000284195	hsa‐miR‐6852	40.13295067
ENSG00000215930	hsa‐miR‐942	39.06812916
ENSG00000221745	hsa‐miR‐1197	38.78852425
ENSG00000207942	hsa‐miR‐136	37.06113531
ENSG00000207944	hsa‐miR‐574	36.46764017
ENSG00000207568	hsa‐miR‐203a	35.70390632
ENSG00000216001	hsa‐miR‐450b	35.41952103
ENSG00000211575	hsa‐miR‐760	33.74964625
ENSG00000221333	hsa‐miR‐548 k	32.79941145
ENSG00000283785	hsa‐miR‐15a	31.76970682
ENSG00000208005	hsa‐miR‐503	29.37422859
ENSG00000199090	hsa‐miR‐326	29.13483177
ENSG00000263456	hsa‐miR‐5189	28.39871136
ENSG00000221754	hsa‐miR‐1260a	25.23317752
ENSG00000265820	hsa‐miR‐3177	24.77186052
ENSG00000263575	hsa‐miR‐4665	24.67230585
ENSG00000207582	hsa‐miR‐30b	23.72914734
ENSG00000283929	hsa‐miR‐5010	23.58517512
ENSG00000221464	hsa‐miR‐1271	23.45850327
ENSG00000207959	hsa‐miR‐656	23.24797065
ENSG00000207698	hsa‐miR‐32	22.99690997
ENSG00000199053	hsa‐miR‐324	22.04029532
ENSG00000207613	hsa‐miR‐181c	21.68525112
ENSG00000211538	hsa‐miR‐501	21.6197488
ENSG00000207996	hsa‐miR‐301a	19.45112604
ENSG00000207730	hsa‐miR‐200b	18.50465325
ENSG00000199005	hsa‐miR‐370	17.84907533
ENSG00000277255	hsa‐miR‐7854	17.66723576
ENSG00000276365	hsa‐miR‐145	16.87146305
ENSG00000199080	hsa‐miR‐133b	16.79696452
ENSG00000283609	hsa‐miR‐4662a	16.70017447
ENSG00000264607	hsa‐miR‐3173	16.28783901
ENSG00000211514	hsa‐miR‐454	15.84741881
ENSG00000283279	hsa‐miR‐376c	15.5124361
ENSG00000263813	hsa‐miR‐3679	15.27222439
ENSG00000284035	hsa‐miR‐5187	15.25222619
ENSG00000199038	hsa‐miR‐210	14.78583447
ENSG00000199012	hsa‐miR‐412	13.67471599
ENSG00000264141	hsa‐miR‐3928	13.43883139
ENSG00000273932	hsa‐miR‐6877	12.95400748
ENSG00000263652	hsa‐miR‐548ax	12.60596835
ENSG00000253008	hsa‐miR‐2355	12.55831531
ENSG00000207754	hsa‐miR‐487b	12.31700799
ENSG00000207617	hsa‐miR‐3074	12.1477769
ENSG00000284154	hsa‐miR‐3605	11.67230493
ENSG00000221463	hsa‐miR‐1277	11.51886948
ENSG00000207988	hsa‐miR‐576	11.43428817
ENSG00000207784	hsa‐miR‐542	10.87185064
ENSG00000267200	hsa‐miR‐132	10.58107814
ENSG00000221025	hsa‐miR‐1250	10.23723849
ENSG00000221510	hsa‐miR‐548o	10.11483819
ENSG00000283203	hsa‐miR‐1246	10.02602246
ENSG00000265565	hsa‐miR‐3143	10.02172938

*Note*: Specifically, the selection here reported was made by including only those miRNAs with average counts >10 copies. The miRNome was performed on four replicates of PL batches. The first 39 miRNAs with a threshold of over 1000 reads are highlighted in red.

Abbreviation: PL, platelet lysate.

As a further selective step, we set a threshold for miRNAs with over 1000 reads, thus obtaining a shortlist of 39 miRNAs (Table 1), which underwent a bioinformatic top‐down analysis on the mirPath v.3 online tool (reverse search). The gene ontology (GO) terms selection was performed according to the function of interest observed for PL treatment on endothelial cells, which is angiogenesis. We interrogated the database by employing five GO, including the first two with the highest hierarchy for processes of capillary and vascular formation, in particular: VASCULOGENESIS (GO_0001570), ANGIOGENESIS (GO_0001525), ENDOTHELIAL CELL ACTIVATION (GO_0042118), VASCULAR DEVELOPMENT (GO_0001944) and ENDOTHELIAL TUBE MORPHOGENESIS (GO_0061154).

After intersecting each GO term with the top 39 miRNA shortlist, we extrapolated potential eligible candidates for the abovementioned roles. We found that the highest overlap was with the angiogenesis gene list as displayed in the function‐expression interaction network that we generated by software (Figure 5C). Afterwards, we compared the results and shortlisted the main group of 31 miRNAs and a further subset of 11 miRNAs correlating with two and all five GO categories, respectively, where three miRNAs (hsa‐miR‐320a, hsa‐miR‐25 and hsa‐miR‐126) were selected to quantitatively validate the seq data by real‐time PCR (Table 2). Notably, miR‐126 is a key regulator of angiogenesis and is known as the angio‐miRNA and one of the most abundant and specific miR to endothelial cells, human platelets and platelet‐derived vesicles.^74^ Notably, the qPCR data accurately highlighted that miR‐126 is the most abundant miRNA in PL, compared to miR‐320a and miR‐25 (Figure 5D; *p* < 0.01 and *p* > 0.001).

**TABLE 2:** List of the 31 miRNAs derived from the bioinformatic analysis to the five angiogenic GOs regarding the shortlist of 39 top expressed miRNAs in PL

	miRNAs	let‐7i	miR‐148a	miR‐26a	miR‐320a	miR‐101	miR‐148b	let‐7g	miR‐199b	let‐7f	miR‐191	miR‐25	miR‐744	miR‐151a	miR‐26b	miR‐185	miR‐30d	miR‐340	miR‐181a	miR‐128	miR‐106b	miR‐127	miR‐28	miR‐140	miR‐98	let‐7c	miR‐126	miR‐186	miR‐152	miR‐16	miR‐30e	miR‐335
GOs	Angiogenesis																															
	Vasculogenesis																															
	Vascularature development																															
	Endothelial cell activation																															
	Endothelial tube morphogenesis																															

*Note*: In bold (black and red) are highlighted the 11 miRNAs included in all five GO categories (each one coloured according to the grayscale). In bold red the three miRNAs (miR‐320a, miR‐25 and miR‐126) employed for the validation of the small RNA seq of all platelet lysate batches. The miRNA belonging to the GO category is coloured differently as displayed. The white box suggests no association to the GO indicated.

Abbreviations: GO, gene ontology; PL, platelet lysate.

**FIGURE 6:**
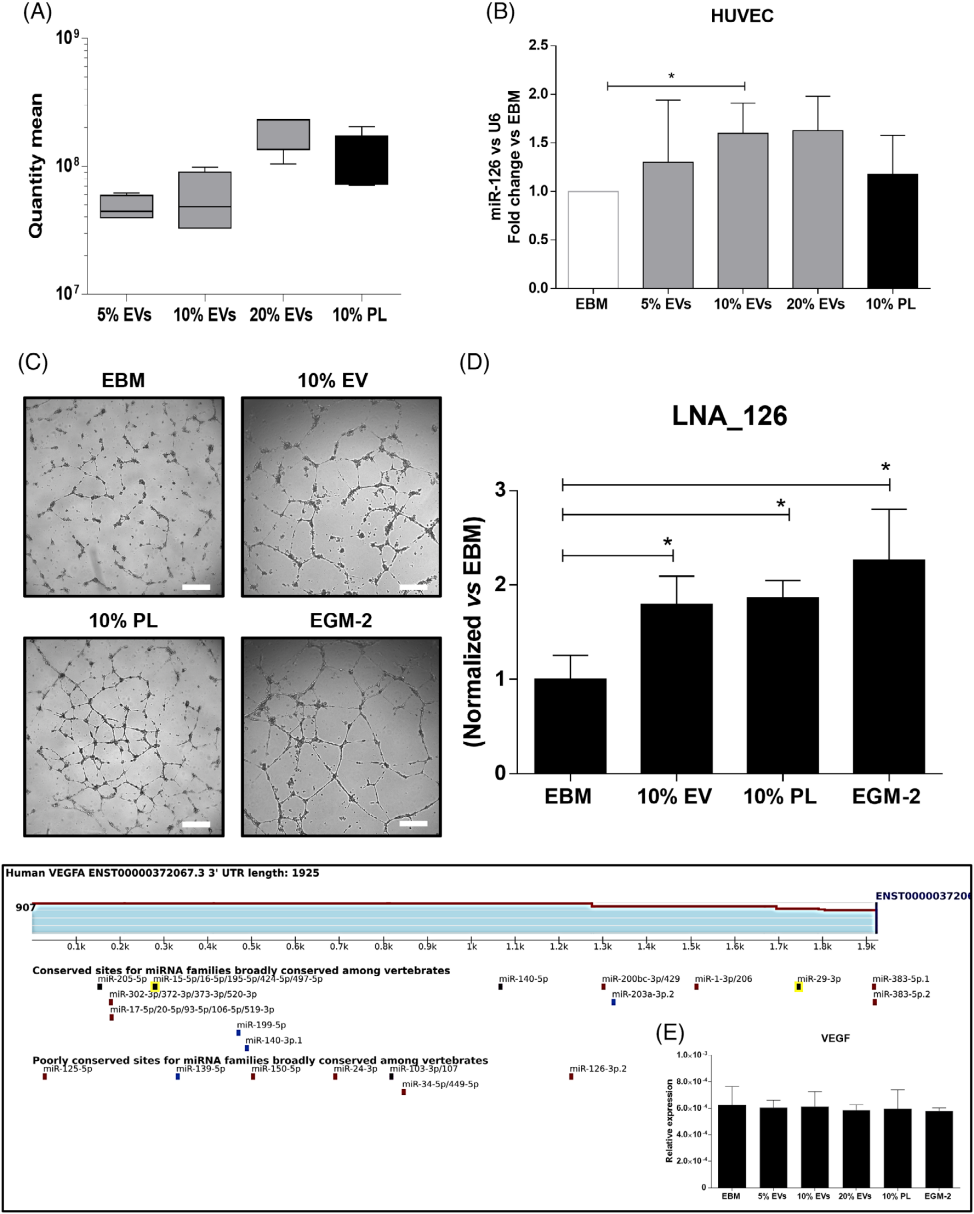
Evaluation of hsa‐miR‐126 in endothelial cells after treatment with PL‐derived EVs. (A) Quantitative real‐time PCR, showing a similar amount of has‐miR‐126 among the percentages of EVs (5%, 10% and 20%) and 10% PL when employed as stimulus. One‐way ANOVA test was applied. A range of *N* = 3–16 experiments was performed. (B) Relative expression of has‐miR‐126 in HUVEC by real‐time PCR after treatment with 5%, 10% and 20% EVs or 10% PL. The fold change on the basal EBM is indicated. *p < 0.05. (C) Representative optical images and (D) quantification of the matrigel assay of HUVEC after antagomir‐126 (LNA_126) transfection and different treatments. Magnification ×4. White scale bar = 200 μm. *p < 0.05. (E) Targetscan analysis at the human 3′‐UTR of VEGF, highlighting that it is a target of miR‐126‐3p in the poorly conserved category and the validation by RTPCR showing no modulatory effect of this gene in our system. A range of *N* = 3–7 experiments was performed. ANOVA, analysis of variance; EV, extracellular vesicle; HUVEC, human umbilical vein endothelial cell; PL, platelet lysate

Next, we validated the role of miR‐126 in the biological effects of PL and EVs on HUVECs, by comparing the sole stimuli that were the media supplemented with 10% PL or 5%, 10% and 20% EVs (with volumes and dilutions adjusted to correspond to 10% PL). Although an increasing but not statistically significant trend of miR‐126 was observed among the percentages of EVs, real‐time PCR testing confirmed the equivalent content of miR‐126 in all media recipes (Figure 6A). The treatment with 10% EVs was the only able to significantly upregulate the levels of intracellular miR‐126 in endothelial cells compared to the negative control (Figure 6B; *p* < 0.05). We sought to verify whether EV‐miR‐126 could play a direct role in mediating angiogenesis ascribable to the transferring of this miRNA through EVs. To this aim, we transfected HUVECs with the antagomiR‐126 (LNA‐126) to rule out endogenous contribution.^75^ Then, we stimulated the cells with 10% EVs or PL. Results showed that HUVECs were efficiently transfected by both the LNA‐126 and control, as shown by flow cytometry analysis (Figure S1a; 84.84 ± 0.4% positive cells with LNA‐126 and 78.5 ± 5.15% with control). Moreover, the copy number of miR‐126 in HUVEC, quantified by droplet digital PCR, was significantly decreased at the lowest levels 24 h after treatment with the LNA‐126, compared to both untreated and transfection controls (Figure S1b; *p* < 0.01 and *p* < 0.05, respectively), while at 48 h the levels had increased again.

Finally, we tested the functional effects of blocking EV‐miR‐126 on angiogenesis and if HIF‐1α or VEGF, known to upregulate miR‐126^76^ and to be abundant in PL,^2^, ^46^, ^47^, ^48^, ^49^ respectively, might be downstream targets. In physiological conditions, the Matrigel assay showed that the number of loops significantly increased in presence of LNA‐126 but with treatment with 10% EV or 10%, PL compared to control EBM (Figure 6C,D, all *p* < 0.05 and Figure S1c,d for Matrigel with control), therefore, confirming the angiogenic effect contained in the EVs derived from PL and mediated at least partially by miR‐126. The analysis of the matches to human 3′‐UTRs of both HIF‐1α and VEGF through the TargetScan software revealed that only VEGF is a target of miR‐126‐3p in the poorly conserved category (Figure 6E). Nevertheless, the validation by real‐time PCR showed no modulatory effect in our system ascribable to VEGF (Figure 6E), suggesting different molecular targets controlled by miR‐126.

## 4 DISCUSSION

This study demonstrates that PL formulations are enriched with EVs, which are biologically active and efficiently sustain angiogenesis in endothelial cells. Interestingly, the EV concentration in our PL is very high and mainly composed of a small EVs subset (50–200 nm),^58^ suggesting that PL might be reinterpreted as an abundant biological source of this EVs subpopulation.

Intriguingly, in line with reports showing comparable in vitro and in vivo haemostatic properties of both platelet microparticles and PL‐derived EVs on platelets,^77^, ^78^, ^79^ our EVs only partially retain this feature. Our PL‐derived EVs do not promote platelet aggregation, but they are able to preserve coagulation in a physiological manner, strengthening their versatile use as angiogenic/antiaggregant mean for cardiovascular applications (where platelet aggregation is a critical risk factor^80^), as a topical product for bleeding during surgery, or as a substitute of platelet transfusion. Anti‐aggregant therapies are acknowledged to negatively impact coagulation, causing bleeding,^81^ therefore, the possibility to investigate and distinguish these two interconnected haemostatic properties in platelet‐derived products (such as PL) is of paramount significance, in order to develop novel products with a unique haemostatic function compatible with the clinical use.

Our results demonstrated that EVs after being internalized by endothelial cells, positively enhances angiogenesis by fostering endothelial tubule‐like networks in a complex 3D microenvironment, similarly to PL. This phenomenon is in line with those describing the angiogenic effects of EVs derived from circulating intact platelets,^82^, ^83^ or from other non‐platelet cell types.^84^, ^85^ Although PL also contains a plethora of several soluble mediators with angiogenic function,^2^, ^46^, ^47^, ^48^, ^49^ it is conceivable that EVs implement this property that PL normally possesses. Notably, 10% of EVs were revealed as the optimal condition in culture, showing a non‐canonical dose–response of EVs without additional effects at a higher percentage in line with the variable biological effects of EVs already verified.^86^ The 10% EVs might represent a sort of ‘balanced’ amount. We have already experienced that the 10% PL itself is the optimal percentage for angiogenic assay also in presence of inhibition of specific soluble factors within the preparation. Below or above this threshold angiogenic effects are not optimal.^46^ Nevertheless, some biological differences exist between PL and EVs: these latter preserve the physiological levels of hydrogen peroxide (<100 μM)^87^ more efficiently than PL and other percentages of EVs in parallel to a comparable redox status (Nox4), therefore generating an anti‐oxidant microenvironment, known to foster beneficial angiogenic effects in endothelial cells^68^, ^87^, ^88^, ^89^ and to preserve vascular function, homeostasis and integrity of the vascular network beyond pathological scenarios.^90^ This phenomenon also occurs in platelets: H_2_O_2_ enhances their activation^91^ or aggregation upon specific agonists,^92^ resulting in a loop of specific NADPH which acts as a sensor of the H_2_O_2_ axis in endothelial cells.^68^, ^93^, ^94^ This result is consistent with the lower production of H_2_O_2_ found between EVs and PL. It is plausible that EVs contain the machinery for both oxidant and anti‐oxidant molecules, therefore acting in a double fashion according to metabolic needs and signals within the microenvironment.^95^

To date, the molecular mechanism by which clinical preparations obtained from platelets enhance regenerative angiogenesis remains not fully explored. Both platelets, the main contributor of miRNAs released in the blood, and their microparticle counterparts, contain a wide range of overlapping miRNAs,^96^ whose investigation so far has been restricted mainly to physiological functions related to aggregation and activation.^96^, ^97^ The miRNAs derived from EVs of platelet origin are both novel biomarkers in the context of anti‐platelet therapies and platelet function,^98^ and biological mediators in the cellular microenvironment,^6^, ^99^, ^100^ suggesting their extra‐platelet role beyond hemostatic properties.

We have demonstrated that half of the small RNA content of PL is composed of miRNAs. We found that angiogenic miRNAs (miR‐320, miR‐25 and miR‐126) are contained in the EV cargo as well as in PL. So far, proper screening of the miRNA profile has been performed only in PRP^101^ and intact or hyperreactive platelets from healthy subjects, or in the presence of cardiovascular pathologies. Interestingly, when we profiled our PL, data have shown that the formulation reflects a similar repertoire of mature miRNAs found in human platelets and described in the literature,^71^, ^72^ including the abundant miRlet‐7 (a marker of platelet differentiation and maturation in megakaryocytopoiesis^102^), or defined microRNA families (i.e., miR‐25 and miR‐103).^72^ By intertwining the transcriptomic expression profile with the vascular function, we have confirmed that in PL‐based preparations the miRNAs quantitatively more represented are also strictly interconnected to the angiogenic function. The EVs contained in PL mirror this picture and confirm that miR‐126 is the most significant miRNA in both preparations.

The angio‐miRNA miR‐126 is one of the most abundant miRNA expressed in platelets.^103^, ^104^ Sharing with miR‐320 (that we also found as highly represented) the unique expression also in endothelial cells, miR‐126 is able to downregulate adhesion molecules (e.g., VCAM‐1) upon the influence of specific cytokines (i.e., VEGF), therefore contributing to endothelial migration, proliferation, activation and vascular inflammation.^75^ Exosomes enriched in miR‐126 are strictly correlated with protection from ischemic events^105^ and atherosclerosis progression.^106^ Changes in circulating levels of miR‐126 have been described in patients with acute ischemic stroke,^107^ coronary artery disease or type 2 diabetes.^108^ Moreover, vascular development and integrity are sustained by miR‐126 in zebrafish and mice,^109^, ^110^ whereas in vivo silencing of miR‐126 impaired angiogenesis^111^ upon ischemic insult. Thus, miR‐126 appears as a potential biomarker and therapeutic target for angiogenesis. Nonetheless, the transfer of miRNAs in the form of EVs under physiological and pathological conditions from platelets to endothelial cells (and vice versa), and the modality by which biological functions are sustained, are still under intensive investigation.

Our data confirm that miR‐126 of platelet origin plays a key role in the angiogenic homeostasis of endothelial cells. Accordingly, results highlight that HUVECs increase intracellular levels of miR‐126 upon stimulation with EVs of PL origin, by adding exogenous miR126 by EV transfer when the endogenous miR‐126 is silenced. Thus, our results demonstrate that a fraction of the angiogenic effect induced by the whole PL preparation is directly ascribable to the EV cargo, specifically to platelet‐derived miR‐126.

This study has some limitations. Although we found that VEGF is a target of miR‐126, we could not observe any modulatory effect in our system, suggesting that alternative mechanisms are needed to be verified. Only a few of them have been already described. For instance, the DNA methyltransferase, playing a role in hypoxia tolerance, has been found as a target of miR‐126 contained in exosomes.^112^ Further mechanisms can coexist, including the reduction of cell apoptosis,^105^ the overactivation of autophagy through Beclin,^113^ the novel delivery system by apoptotic bodies through CXCL12^114^ or the inhibition of the negative regulators of the VEGF‐axis.^110^, ^115^ More importantly, the angiogenic properties of both EVs and PL cannot be explained uniquely by the miR‐126. The miRNome here described suggests the presence of additional miRNAs with similar functions. For instance, the exosomal derived‐miR25 has been found to promote angiogenesis, vascular permeabilization, metastatic niches in cancer and involvment in cardiovascular disorders.^116^, ^117^

To date, the individual contribution of EVs within PL has not been fully elucidated in terms of regenerative angiogenesis. Certainly, the methodology to manufacture PL severely impacts the quantity and the quality of EVs within the formulations and in particular that employed to concentrate, lyse or activate platelets^118^ in the formulation. Accordingly, PL preparations with excessive heterogeneity of EV content might result in parallel different downstream signalling and pathways activated with a wide range of unpredictable biological effects, also depending on cells potentially targeted by clinical PL preparations.

In conclusion, PL‐based formulations are a source of both biologically available miRNAs and EVs defining the hallmark of platelet origin. The EVs reflect the ‘angiogenic physiology’ of PL, confirming that a cell‐free therapy approach may be a novel effective strategic tool in clinical applications.

Future investigations will be required to unveil the role of downstream targets of different miRNAs potentially preserved in EVs of platelet origin, and how additional processes not limited to angiogenesis are modulated, including immunomodulatory functions and paracrine effects.

## AUTHOR CONTRIBUTIONS

Antonella Bordin performed the main experiments. Maila Chirivì, Marika Milan and Roberto Rizzi performed 3D constructs. Francesca Pagano performed qPCR of small RNA seq. Marco Iuliano and Eleonora Scaccia isolated the EVs. Orazio Fortunato and Giorgio Mangino performed NTA and cytofluorimetry. Xhulio Dhori developed the matrix for the angiogenic network of the small RNA seq. Elisabetta De Marinis performed the droplet digital PCR. Alessandra D'Amico and Fabio Pulcinelli performed all experiments on aggregation and oxidative states. Selenia Miglietta performed the TEM. Vittorio Picchio the cell transfection. Giacomo Frati and Isotta Chimenti reviewed and edited the manuscript. Elena De Falco conceived the study and wrote the paper.

## CONFLICT OF INTEREST

The authors declare that they have no conflict of interest.

## Supporting information


**FIGURE S1** Validation of cell transfection with antagomir‐126 (LNA_126) in endothelial cells. (A) Representative histogram of the cytometric analysis of HUVEC with LNA_126 (light blue line) and Control (orange line) in FITC channel. Autofluorescence is highlighted with the red line. (B) Absolute quantification of hsa‐miR‐126 by Droplet digital PCR, showing the efficient downregulation of the number of the copies in HUVEC after transfection with LNA_126 LNA or Control (Ctl_126) compared to untreated cells (WT) at 24 h and 48 h. (C) Representative optical images and (D) quantification of the matrigel assay of HUVEC after Control (Ctl_126) transfection and different treatments. Magnification 4X. White scale bar, 200 μm. *p < 0.05.

## Data Availability

Main data generated or analysed during this study are included in this article, and detailed data are available from the corresponding authors on reasonable request.
