# Supplementary figures and images for: Human platelet lysate‐derived extracellular vesicles enhance angiogenesis through miR‐126
Source: Cell Prolif. 2022 Aug 9;55(11):e13312. doi: 10.1111/cpr.13312 (PMC9628251; doi:10.1111/cpr.13312)

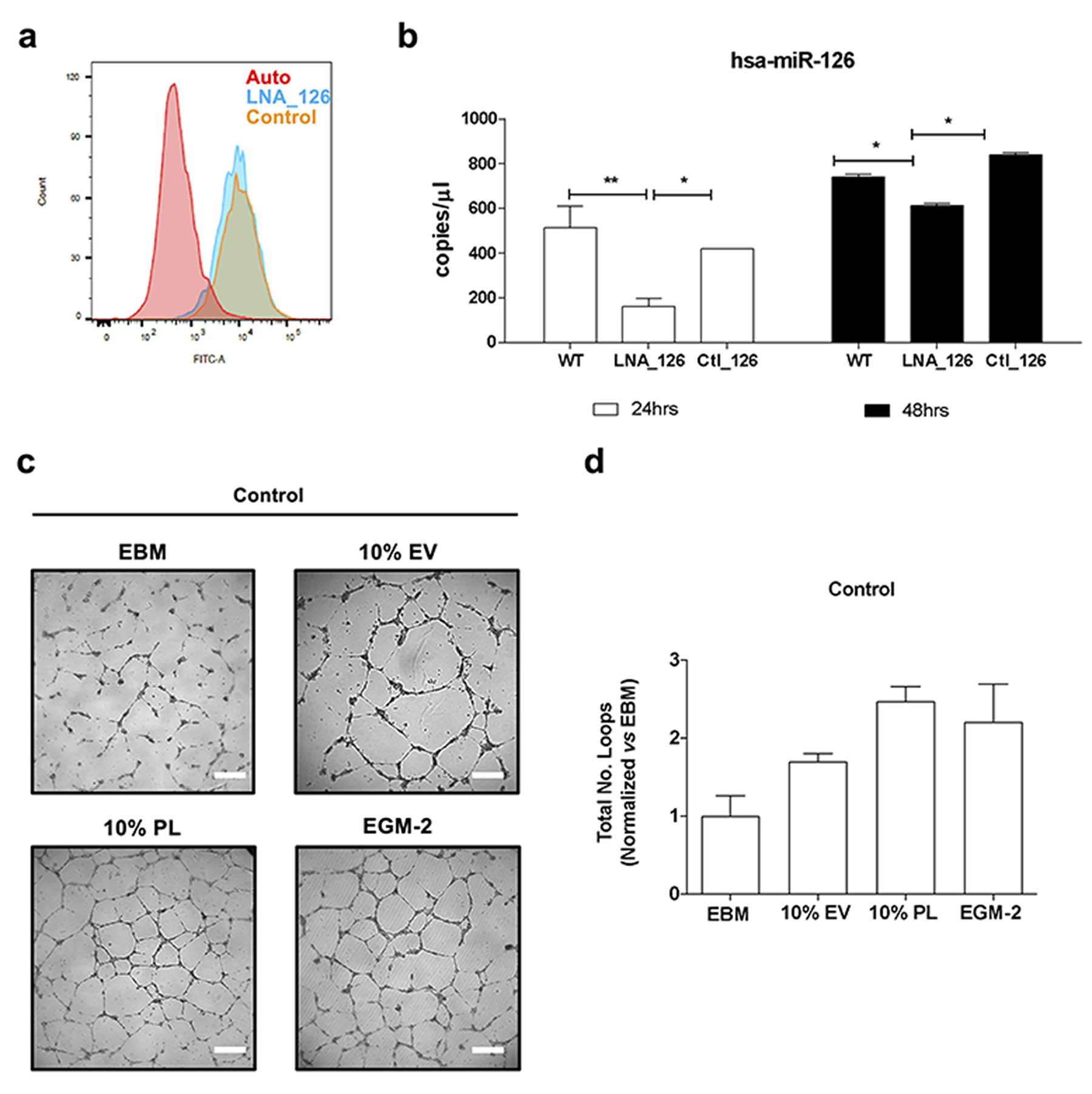

Supplement: Supplementary file 1 — FIGURE S1 Validation of cell transfection with antagomir‐126 (LNA_126) in endothelial cells. (A) Representative histogram of the cytometric analysis of HUVEC with LNA_126 (light blue line) and Control (orange line) in FITC channel. Autofluorescence is highlighted with the red line. (B) Absolute quantification of hsa‐miR‐126 by Droplet digital PCR, showing the efficient downregulation of the number of the copies in HUVEC after transfection with LNA_126 LNA or Control (Ctl_126) compared to untreated cells (WT) at 24 h and 48 h. (C) Representative optical images and (D) quantification of the matrigel assay of HUVEC after Control (Ctl_126) transfection and different treatments. Magnification 4X. White scale bar, 200 μm. *p < 0.05. [file CPR-55-e13312-s001.tif]
